# Chromidrosis

**Published:** 1902-03-15

**Authors:** Geo. Parker

**Affiliations:** Physician, Bristol General Hospital


### Hospital Clinics and Medical Progress.

### CHROMIDROSIS.

By Geo. Parker, M.A., M.D., Physician, Bristol General Hospital.

Real instances of this affection are very rarely seen. Indeed only about forty-five cases are on record, though the malingerer and the neurotic woman not infrequently present good imitations. If we include under the name only cases of pigmented sweat or sebum it will be necessary to put on one side numerous forms of mere pigmentation of the skin, such as the spots or patches in Addison's disease, arthritis deformans, chloasma, xanthoma, freckles, and the stain left after certain syphilitic eruptions, lichen, and other diseases, as well as the colour caused by obstructive jaundice, or by the administration of nitrate of silver in excess. Here, too, may be added the yellowish-brown tint seen in some forms of kidney and liver disease, and the pale yellow which accompanies many forms of hæmolysis, though, indeed, some of these may finally prove to be the result of the secretion of certain pigment-forming bodies in the sweat. In the majority of these affections the pigment appears to be either melanin or some product of the colouring matter of the blood, and in either case it stains the epithelial tissues and is not simply deposited on the surface.

There is yet another group of affections to be distinguished from chromidrosis. Here we find an exudation of blood serum, or of a liquid which has been supposed to be urine from the surface of the skin and occasionally from the ducts of the sebaceous or sweat glands. Such appearances have often been due to imposture, but there are plenty of instances of extraordinary hæmorrhages even from the gland ducts themselves, as in a case where Hebra observed a tiny stream of blood flow from a sweat gland on the back of the hand. The much discussed stigmata on the hands and feet of various enthusiasts and hysterical persons have often been as genuine as the hæmorrhages in purpura, but the bleeding is not of the nature of sweat. The Belgian girl who attained such fame some years ago by her weekly bleedings showed first the development of a large vesicle or bulla, after which followed the hæmorrhage in the course of a few hours.

Another remarkable class of cases depends on the infection of the sweat after it is excreted by some colour-forming organism. Thus red sweat is sometimes produced by a growth of bacillus prodigiosus in the armpits and elsewhere. The growth may be so profuse that not only are the hairs encrusted by it but the sweat itself may be tinged. It is sometimes most difficult to determine the cause of the phenomenon. Thus, in a case recently reported a bright blue mark appeared between each of the toes on both feet, in a married woman, the mother of ten children. This first occurred during an attack of rheumatic fever, and lasted six months, in spite of various applications. It then faded away, to reappear with a return of the rheumatism some months later. Neither ether, chloroform, nor ammonia could remove the pigment, and the question arose whether the stain was due to a growth of the bacillus pyocyaneus, or to some constituents of the sweat, or to accidental and undetected aniline or copper pigment derived from the clothing. Purdon mentions a similar blue pigmentation in a rheumatic patient of middle age, and Speranza a green one, also accompanied with rheumatism, in a girl of fourteen years.

The form of chromidrosis which has been most fully investigated by a committee of the Clinical Society and others is in some way connected with the formation of indican in the intestine. The patients generally suffer from constipation; they are usually young women of a neurotic type, or at least sufferers from menstrual disorders. The pigment has been collected and analysed and found to contain besides other matters certain dark-blue granules of which the exact composition is uncertain. The sweat is dark brown, black, or bluish, and is chiefly found on the forehead, face, and especially the lower eyelids. It is worth remarking that pigmentation in the latter situation during menstrual disorders is very common. Various precautions to prevent fraud have been taken by different observers, such as covering a clean area of skin with a watch glass and placing the patient in a Turkish bath, and in at least one case a spot of pigment was seen to form and extend while under direct and continued observation, so that there is no doubt of the reality of the affection. The material can be wiped or washed off in considerable quantities, and is formed as rapidly as ordinary sweat, thus in Brodie's case the upper half of the face became as dark as a negro's in a few hours. The skin itself appears quite normal, or at most a little tender. Sometimes the pigment is also observed in the urine, saliva, or vomited matters as well as in the sweat and sebum. Besides these instances of dark sweat others have been found of a yellow, pink, green, or red colour. The hands, feet, or trunk may occasionally be affected, and just as hyperidriosis may affect one side of the body, so coloured sweat may be limited to one hand, one side of the body, or even to one side of the scrotum. Great caution should, however, be used in diagnosing such affections from the fraudulent artifices of hysterical patients. Finally, the administration of certain drugs will occasionally produce a true pigmentation of the sweat. Pringle mentions a case where iodide of potash produced a pink sweat, staining the handkerchief, hair, and beard of the patient as long as he took it. Iron and copper, and perhaps santonin, have apparently acted as chromogens in rare instances. The wonder is that so few colouring agents are excreted in the sweat. Thus methylene blue gives the urine its extraordinary colour in a few hours, but has little effect on the sweat, neither staining the clothes or colouring the skin, though the mucous membrane of the mouth may be tinged. One of the aniline dyes, which is used as marking ink, has caused considerable alarm by its easy absorption through the skin of infants, and its power of causing a general dark pigmentation over the whole body, lasting for some days without any necessary injury to the health of the child. Several cases have been observed of this kind, and indeed the dusky cyanotic pigmentation can be produced at pleasure by wrapping an infant in linen marked with the dye. We are not aware that any explanation of the colour has been given, and as to whether it is excreted by the glands or how it is formed, but the amount of dye absorbed from the letters written on the linen must be minute.

